# In Vitro Immune Organs-on-Chip for Drug Development: A Review

**DOI:** 10.3390/pharmaceutics10040278

**Published:** 2018-12-15

**Authors:** Aya Shanti, Jeremy Teo, Cesare Stefanini

**Affiliations:** 1 Department of Biomedical Engineering, Khalifa University of Science and Technology, Abu Dhabi, P.O. Box 127788, UAE; aya.shanti@ku.ac.ae; 2 Division of Engineering, New York University Abu Dhabi, Abu Dhabi, P.O. Box 129188, UAE; jeremy.teo@nyu.edu; 3 Department of Mechanical and Aerospace Engineering, New York University, New York City, P.O. Box 903, NY 10276-0903, USA; 4 Healthcare Engineering Innovation Center, Khalifa University of Science and Technology, Abu Dhabi, P.O. Box 127788, UAE

**Keywords:** drug development, drug discovery, immune system, in vitro, organ-on-chip, biomimicry

## Abstract

The current drug development practice lacks reliable and sensitive techniques to evaluate the immunotoxicity of drug candidates, i.e., their effect on the human immune system. This, in part, has resulted in a high attrition rate for novel drugs candidates. Organ-on-chip devices have emerged as key tools that permit the study of human physiology in controlled in vivo simulating environments. Furthermore, there has been a growing interest in developing the so called “body-on-chip” devices to better predict the systemic effects of drug candidates. This review describes existing biomimetic immune organs-on-chip, highlights their physiological relevance to drug development and discovery and emphasizes the need for developing comprehensive immune system-on-chip models. Such immune models can enhance the performance of novel drug candidates during clinical trials and contribute to reducing the high attrition rate as well as the high cost associated with drug development.

## 1. Introduction

The process of pharmaceutical drug development is typically long and expensive, taking up to twelve years of research effort and costing on average more than 1.7 billion US dollars per clinically applicable drug [1,2]. For a novel drug to be approved by the food and drug administration (FDA), it should pass the preclinical evaluation phase, which relies on in vitro cell culture platforms and on in vivo animal models, and the clinical evaluation phase which involves controlled drug administration on human subjects. Unfortunately, many drugs that pass preclinical evaluation fail in clinical evaluation, leaving the pharmaceutical drug development industry with a high attrition rate for novel drug candidates [3,4]. It is estimated that only 1 out of 10,000 new chemical entities gains FDA approval and makes it into the market [5]. One key aspect along the drug development chain that has been overlooked is the effect of the drug candidate on our immune system. In-depth investigations into this aspect are lacking and could unlock significant knowledge to economize the high cost of drug development as well as to reduce the attrition rate, in particular through the mechanistic understanding of the human immune physiology to drugs. However, the current drug development practice lacks reliable and sensitive techniques to evaluate the immunotoxicity of drug candidates at an early stage in a preclinical setting [6]. Existing in vitro platforms often lack physiological relevance as well as microenvironment complexity and thus undermine the intricate in vivo cell-cell and cell-matrix interactions essential for regulating cell behavior [6,7]. On the other hand, although more physiologically relevant, animal models fall far short of being able to predict human immune responses to pharmaceutical drugs due to the intrinsic differences between humans and members of other species [8]. Mouse models are often employed to study immunology and assess the toxicity of newly developed drugs [9]. However, there has been noteworthy differences between the immunity of mice and humans, particularly in terms of the balance of leukocyte subsets, defensins, Toll-like receptors, antibody subsets, T-cell signaling pathway components and chemokine and chemokine receptor expression [9,10]. In addition, in many cases, mice models do not closely mimic diseases including autoimmunity [11]. Furthermore, animal models tend to exhibit low reproducibility and poor controllability over physiological parameters [6]. All these factors have shifted scientific attention towards developing more reliable, accurate and controllable in vitro immune culture platforms for predicting drugs interaction with the immune system and their subsequent safety at an early time point during the preclinical evaluation phase. 

Mechanistic studies of cellular systems are best-performed using experimental setups that closely mimic the native microenvironment of cells. Such controlled setups are possible with microfluidic technology, which has been widely employed in biological research to reduce cost of reagents, maximize information from precious samples and simultaneously provide spatio-temporal information about the dynamics of cells in their microenvironment [12]. The latest advancement in this field has led to the development of the so called “organs-on chip”, which are perfused microfluidic devices consisting of one or more micro-meter sized chambers wherein living cells are cultured and maintained, simulating the physiological function of tissues and organs [13]. Such chips replicate three key features of a tissue or organ: (1) its 3D microarchitecture i.e., the spatial distribution of different cell types (2) its complex biochemical microenvironment including chemokine, growth factor and nutrient gradients and (3) its mechanical microenvironment including mechanical compression, cyclic strain and shear stress [14,15,16,17,18]. Multiple microfluidic chips can be linked together via microfluidic channels mimicking the interaction between different human organs. 

Microfluidic organs-on-chip can be fabricated using various manufacturing techniques including micromolding, microetching, soft lithography and photo-polymerization. These technological processes have enabled the development of fine structures that closely resemble those in vivo [19,20]. Furthermore, powerful computational techniques that allow the quantification of cellular behavior even at the single cell level are now being available and can be combined with microfluidic technology to provide a comprehensive description of cellular behavior under various stimuli [21,22,23].

Over the past decade, many researchers have utilized organs-on-chips in order to evaluate the efficacy and toxicity of drugs in a more in vivo simulating environment [13]. Specifically, many in vitro immune organs-on-chip have been developed. These permit the evaluation of the human immune response to pharmaceutical drugs in a physiologically relevant manner, thus reducing the time and cost associated with bringing novel drugs to the market [24]. In addition, they help improve our understanding of disease physiology and mechanisms including the development of cancer and autoimmune diseases [25]. This review describes existing biomimetic immune organs on chip, their design, and their physiological relevance to drug development and discovery. In particular, it highlights models of the skin, gut, liver, spleen, lymph node, bone marrow and lymphatic vessels. Furthermore, it discusses challenges associated with the development of such immune organs-on-chip and offers a perspective on the importance of developing an integrated model of the whole human immune system.

## 2. Immune Organs-on-Chip

The immune system comprises a network of cells, tissues and organs that function together to protect the body against pathogens such as bacteria and viruses. Immune cells originate in the bone marrow where hematopoietic stem cells develop into common precursor cells that ultimately differentiate into immune cells. Common myeloid precursors give rise to different granulocytes such as neutrophils and basophils as well as to antigen presenting cells such as dendritic cells and macrophages which all circulate throughout the body and scan tissues for foreign antigens [26,27]. On the other hand, common lymphoid precursors give rise to B and T lymphocytes which display receptors that specifically bind to certain antigens. T and B lymphocytes play crucial roles in adaptive immunity due to their ability to retain immunological memory that allows them to initiate fast and specific responses against pathogens [26]. Most B cells develop in the bone marrow and then migrate to secondary lymphoid organs, namely the lymph nodes and spleen, until they receive appropriate signals for activation. T-cell precursors leave the bone marrow and enter the thymus for positive and negative selection to ensure that self-reacting cells are eliminated. Mature T-cells then migrate to the lymph nodes or spleen until they are activated. The activation of T and B lymphocytes requires interaction with an antigen matching the specificity of the receptor expressed on the surface of T or B cells [26]. This interaction usually takes place within the lymph node or spleen and is mediated by antigen presenting cells.

It has been shown that the maintenance of immune homeostasis involves the coordination between immune components localized in various tissues besides lymphoid microenvironments [28]. One such example is the localization of Immunoglobulin-A producing plasma cells, their precursors and the T-cells required for their maturation in gut associated lymphoid tissues in close proximity to the gut lumen where immunoglobulin-A is intended to function [29,30]. Another example lies in the skin which contains characteristic lymphoid components in a microenvironment suitable for specific recognition of and response to foreign antigens [28,31]. Therefore, it is widely accepted today that the main immune organs are skin, gut, thymus, bone marrow, spleen, liver and lymph node [32,33].

**Figure 1:**
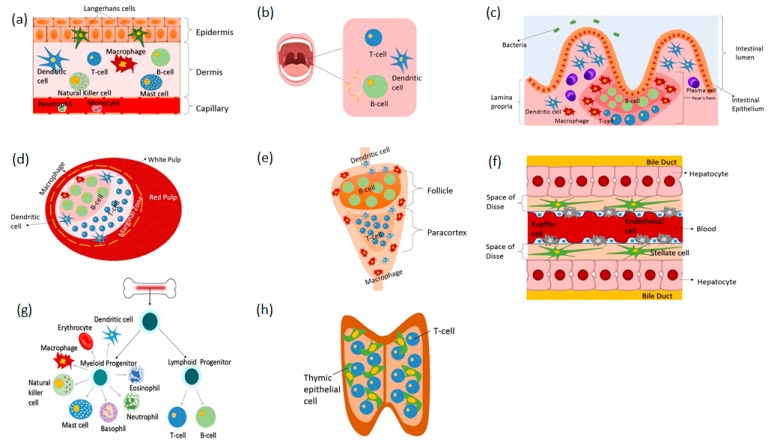
Organs of the immune system and their associated immune cellular components. (**a**) skin (**b**) tonsils (**c**) gut (**d**) spleen (**e**) lymph node (**f**) liver (**g**) bone marrow and (**h**) thymus.

**Figure 2:**
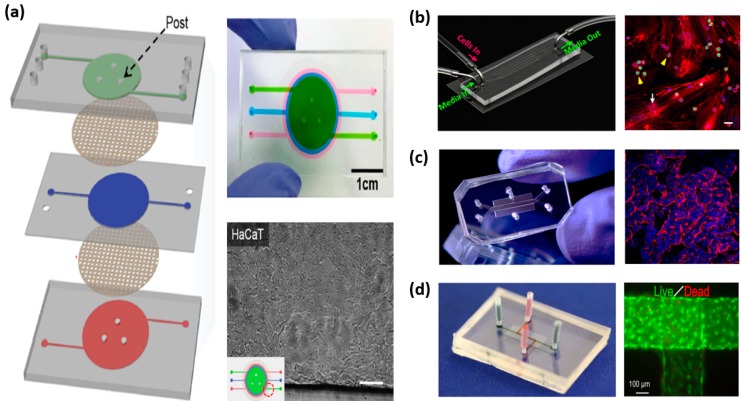
Examples of immune organs-on chip. (**a**) skin-on-chip consisting of three PDMS layers and two porous membranes filled with fluid three different colors or containing HaCaT cells [39], (**b**) bone marrow-on-chip consisting of 4 microchannels and containing SUP-B15 and bone marrow derived stem cell co-culture [40], (**c**) gut-on-chip consisting of two hollow microchannels separated by a porous membrane containing villus epithelium [41] and (**d**) lymphatic vessel-on-chip with a specific microchannel pattern containing blood vascular endothelial cells and lymphatic endothelial cells [42].

All types and all classes of pharmaceutical drugs perturb the immune system, but the mechanisms and immunological outcomes of these perturbations are not well defined [34]. Lack of understanding on these biological-pharmaceutical interactions confounds drug development, conceals potential hidden side effects and limits discovery [34]. Various methods have been employed to study the immune system in health and in disease as well as in response to pharmaceutical drugs. These include in vitro and in vivo experimental studies and in silico modeling techniques [25,35,36,37,38]. In the following, we describe existing in vitro immune organs on chips that can facilitate investigations into the human immune physiology to pharmaceutical drugs. Figure 1 shows the various immune organs of the body. Not all organs have been simulated in vitro, thus, only those having equivalent models on-chip will be discussed in this review. More specifically, the skin, gut, liver, spleen, lymph node, bone marrow and lymphatic vessel models will be discussed. Figure 2 shows examples of immune organs-on-chip developed by various researchers.

### 2.1. Skin-on-Chip

The skin is not only the largest organ of the human body but also the first organ of defense protecting against invading microorganisms and other pathogens. Besides acting as a physical barrier separating the internal organs of the body from the external environment, it is an active immune organ containing elements of both the innate and adaptive immune systems [43]. Immunity within the skin lies is in its two major layers, the epidermis and the dermis. The key immune cells located in the epidermis include (1) keratinocytes which express Toll-like receptors that detect conserved molecules on pathogens and (2) Langerhans cells which are specialized antigen presenting DCs [44,45]. The key immune cells located in the dermis include (1) mast cells (2) DCs and (3) T lymphocytes [44]. Upon pathogen encounter, epidermal immune cells secrete vast amounts of anti-microbial peptides, proinflammatory cytokines and chemokines which are consequently absorbed by the dermis [46,47]. This activates the immunocompetent cells of the dermal layer, triggering an immune response. 

Many skin-on-chip platforms have been developed to aid in the process of drug development and testing. These vary in the level of complexity, the availability of vascular elements, and the types of cells they incorporate. For instance, O’Neill et al. developed a skin-on-chip model that simulates the outermost epidermal layer of the skin only. It consists of neonatal human epidermal keratinocytes cultured on collagen patches in microfluidic channels [48]. This model is a simplistic representation of the epidermis, much like a keratinocyte culture system. In a more recent attempt, Sriram et al. developed a full thickness skin on chip model consisting of both a dermal equivalent and an epidermal one [49]. The dermal equivalent is a fibrin-based matrix seeded with human primary foreskin-derived dermal fibroblasts while the epidermal equivalent is a keratinocyte generated epidermis formed by culturing N/TERT-1 keratinocytes over the apical surface of the dermal equivalent. N/TERT keratinocyte cell lines have been shown to be good alternatives for human primary keratinocytes in epidermal studies. The model is double side perfused (from apical and basal layers) and supports both liquid to liquid interface and air to liquid interface. The integration of air to liquid interface into the skin model is beneficial and was shown to be essential for the maturation and differentiation of human epidermal keratinocytes [50]. 

To achieve more physiological relevance, several studies have also incorporated vascularization into their skin-on-chip models utilizing primarily endothelial cells [39,51,52]. Mori and colleagues developed a perfused full-thickness skin model consisting of both an epidermal and a dermal equivalent whereby the dermal equivalent is penetrated by nylon wires lined by Human umbilical vein endothelial cells (HUVEC). To demonstrate the feasibility of using this model for drug development, the authors measured percutaneous absorption of Isosorbide dinitrate and caffeine as model drugs and showed their differential permeation. Wufuer and colleagues also developed a vascularized skin-on-chip model but to simulate inflammation, edema and drug based treatment [39]. The model consists of three Polydimethylsiloxane (PDMS) layers stacked on top of one another on which human keratinocytes, HS27 fibroblasts, and HUVECs were seeded replicating the epidermal, dermal and vascular layers respectively. The layers were separated from one another using transparent, porous polyester membranes allowing interlayer communication and mimicking skin biology. The porous membranes allow the diffusion of molecules such as nutrients, cytokines and drugs between the different layers. Similarly, Abaci et al. developed a vascularized human skin equivalent but utilizing induced pluripotent stem cell (iPSC) derived endothelial cells. The authors demonstrates that the iPSC-derived endothelial cells promote host neovascularization and induce vessel invasion from the wound bed towards the epidermis [51].

All the previously reported vascularized skin-on-chip models lack key immune cells such dendritic cells and T-lymphocytes, thus undermine the immunocompetence of skin. The only available model that incorporates immune elements is one developed by Ramadan and Ting [53]. The model comprises a layer of keratinocytes (HaCat) and a layer of monocytes (U937) separated by a porous membrane that facilitates inter-communication in a bi-channel microfluidic setting. The authors demonstrated the ability of the model to respond to external stimuli, both chemical and physical, by using Lipopolysaccharide (LPS) and UV stimulation respectively. The response was assessed through trans-epithelial electrical resistance measurement and magnetic bead-based sandwich immune assay. Furthermore, the authors show that the expression of the pro-inflammatory cytokines IL-6 and IL-1β in response to LPS stimulation in the developed skin model is different than that of keratinocyte monoculture or monocyte monoculture, highlighting the importance of incorporating immune elements into skin models and in substance or drug testing.

### 2.2. Gut-on-Chip

Similar to the skin, the lining of the gastrointestinal (GI) tract is exposed to the external environment and therefore, it constantly encounters pathogens [54]. This requires the GI tract to maintain high fidelity defense mechanisms against invading pathogens in order to prevent their entry into internal organs of the body. In fact, the GI tract is home to an enormous amount of structures with immune cells that help maintain immune homeostasis in the face of pathogenic challenges [55]. Epithelial cells provide physical separation between the lumen of the stomach as well as of the intestine and the underlying tissues. Mucous cells, on the other hand, maintain a luminal mucosal layer that prevents contact between epithelial cells and pathogens. Beneath the epithelial layer reside white blood cells that can effectively respond to perturbations, inducing controlled inflammatory responses at certain occasions and inhibiting them at others [55,56,57]. Specifically, small masses of lymphocyte-rich tissues known as Peyer’s patches are situated throughout the ileum region of the small intestine to monitor intestinal bacteria populations and to prevent the growth of pathogenic ones. Furthermore, the GI tract houses a microbial community which has been shown to live and interact with gut lymphoid tissues as well as regulate the development and functionality of the immune system [58,59]. All these factors make the GI tract an important component of immunity that needs to be addressed during drug development and discovery, especially in the case of orally administered drugs which come in close proximity to the defense mechanisms of the GI tract.

An efficient micro-engineered gut-on-chip should incorporate the key features of the GI tract which include: an epithelial barrier, a mucus layer, an immune component and a human-microbial interface. It should also support peristalsis like motion and the transport of nutrients or agents through the epithelium. Many of these features have been addressed by researchers in literature but to a variable extent and for different applications. 

Intestine-on chip platforms vary in their complexity and functionality. The earliest and most simple model was developed by Kimura et al. to support long-term culture and monitoring of epithelial colorectal adenocarcinoma cells (Caco-2), which are common cellular models for examining small intestinal functions [60]. It consists of two independent channels separated by a polyester, porous, semipermeable membrane on which Caco-2 cells are cultured. The device is equipped with a micropump to circulate culture media in a pulsatile manner and it is integrated with an optical detection system. The authors showed that the cells could be cultured for more than thirty days and form a functional epithelial monolayer supporting substance transport activities as evident by on-line fluorescent measurements of rhodamine-123. Using a similar setup and the same cell line, Kim et al. developed a more in vivo simulating environment of the intestine which involves biophysical cues, specifically, shear stress and cyclic strain [61]. In addition, it incorporates microbial flora that have been shown to be necessary for normal intestinal function [62,63,64]. The authors demonstrated that low levels of fluid flow and shear stress promote accelerated formation of columnar epithelium which polarizes rapidly and develops 3D villi-like structures. The differentiated epithelium exhibits increased intestinal barrier function and supports the growth of bacteria Lactobacillus rhamnosus GG that normally inhabits the human intestine. Still, the addition of cyclic strain enhances these responses. Further extending the results, Kim and Ingber utilized that same gut-on chip model with its peristalsis generating capability to examine the morphogenesis of the polarized epithelium and the subsequent formation of villus-like structures [65]. They showed that the cells lining the villus structures are of four differentiated subtypes of epithelial cells; absorptive, mucus-secretory, enteroendocrine, and Paneth and that they adopt positions similar to those observed in vivo. Additionally, they demonstrated that the cells lining the villus structures are linked together via well-defined tight junctions at their intercellular boundaries along their apical surfaces and covered by brush borders as well as by mucus. Moreover, they indicated that the process of villus formation resulted in a 1.7-fold increase in exposed apical cell surface area compared to that of the monolayer of cells. Interestingly, the cells cultured in the gut-on chip device displayed progressive increase in the activity of a drug metabolizing cytochrome enzyme during the first hours of culture while the cells cultured in monolayer within a Transwell insert failed to exhibit any significant cytochrome activity, which highlights the importance of developing models that closely mimic the native microenvironment of cells especially for pharmaceutical drug development applications.. To further demonstrate the versatility of this platform and its potential to analyze intestinal pathophysiology as well as drug outcomes, Kim et al. utilized the gut-on chip to examine how probiotic and pathogenic bacteria, LPS, immune cells, inflammatory cytokines, and mechanical forces contribute individually and collectively to intestinal inflammation, villus injury, and integrity of the epithelial barrier [66]. In this setup, they incorporated eight strains of probiotic bacteria, many of which are derived from human microbial gut, a strain of pathogenic Escherichia coli and immune cells in the form of peripheral blood mononuclear cells (PBMCs) from healthy donors. The results indicate that the lack of peristalsis-like motion and the subsequent epithelial deformation initiates bacterial overgrowth similar to that observed in patients with ileus and inflammatory bowel disease. In addition, the simultaneous presence of immune cells and LPS triggers the production of four proinflammatory cytokines, IL-8, IL-6, IL-1β, and TNF-α, by epithelial cells which together induce villus injury and compromise the intestinal barrier function. Finally, probiotic and antibiotic therapies were shown to suppress villus injury induced by pathogenic bacteria. 

Kim et al.’s gut-on chip is not the only available on-chip model of the intestine; although it is the most physiologically relevant and the most versatile. Shim et al. similarly developed a gut-on chip but with villi-like structures fabricated as part of the microfluidic device rather than forming as a result of physiological cues [67]. The results revealed that the morphology and functionality of the epithelial cells are enhanced compared to those cultured in monolayers. In addition, Shah et al. developed a microfluidic model to capture the gastrointestinal human–microbe interface [68]. Taken together, all these models are valuable platforms that make it possible to examine the human physiology and its subsequent alterations in response to external stimuli being it in the form of pathogens or even pharmaceutical drugs. Certainly, such gut-on chip platforms can be exploited to assess the interplay between the immune components and drugs. 

### 2.3. Liver-on-Chip

Although not usually perceived as an immune organ, the liver has been shown to possess key immunological properties such as the incorporation of a diverse population of resident immune cells including the body’s largest population of resident macrophages known as Kupffer cells as well as the production of acute phase proteins, complement components, cytokines and chemokines [69,70]. Kupffer cells are localized within the lumen of hepatic sinusoids and play a significant role in innate immunity. They constantly encounter gut-derived bacteria, debris and endotoxins [71]. Subsequently, they get activated. Upon activation, they secrete various products including cytokines, prostanoides, nitric oxide and reactive oxygen species [72]. These products in turn, regulate the phenotype of Kupffer cells as well as that of hepatocytes, stellate cells, endothelial cells and other immune cells that traffic through the liver. This way, Kupffer cells prevent the movement of immunoreactive substances from the GI tract past the hepatic sinusoid and regulate the liver response to infection, to toxins and to other stresses including pharmaceutical drugs [72,73].

Many liver-on-chip platforms have been developed to support the prolonged culture of liver cells and to facilitate investigations into the various functions of the liver including metabolism, detoxification and response to pharmaceutical drugs [74,75,76,77,78,79,80,81,82,83,84,85]. For instance, Prodanov et al. developed a microfluidic device consisting of two chambers separated by a porous membrane capable of maintaining hepatocyte culture for up to 28 days [86]. Similarly, Kang et al. developed a microfluidic system containing coculture of hepatocytes and endothelial cells in both single and dual microchannel configurations, both with and without continuous perfusion and utilized the system to analyze viral replication of hepatotropic hepatitis B virus [87,88]. The authors demonstrated that hepatocytes maintained their normal morphology for at least 30 days and continued to produce urea for the whole duration. In addition, they showed that Hepatitis B virus replication was successfully detected in the system. In a different application and in order to demonstrate the utility of such systems for drug screening, Khetani and Bhatia developed a microfluidic culture system for hepatocyte and hepatocyte-fibroblast coculture with patterned extracellular matrix and used it to quantify the acute and chronic toxicity of model hepatotoxins such as troglitazone [84]. The authors showed that troglitazone caused significant morphological changes in hepatocytes followed by extensive cell death after up to 9 days of exposure. Interestingly, none of the existent liver-on-chip models examined the immune competency of the liver and its role in drug rejection. In fact, drug toxicity to the liver is the most common cause for the discontinuation of clinical trials on a drug, as well as the most common reason for an approved withdrawal of a drug from the marketplace [89]. This is, in part, due to the underestimation of liver-immune cellular interactions. If models of the liver containing key immune components such as Kupffer cells are developed, they would contribute significantly to our understanding of liver disease mechanisms as well as of immune physiology to drugs.

### 2.4. Lymph Node-on-Chip

Immune responses initiated at different locations throughout the body including the skin and gut are coordinated by secondary lymphoid organs, mainly the lymph nodes, which are situated at strategic locations throughout the body. Lymph nodes are cellular microenvironments for immune cells. They collect draining interstitial fluid that flows across all tissues accumulating foreign particles, distributed pharmaceutical drugs as well as immune cells that congregate there for intracellular communication purposes [90,91]. Within the lymph nodes, B and T lymphocytes scan this fluid for the presence of foreign antigens, that once recognized are effectively eliminated from the body. The lymph nodes provide an optimal environment for the lymphocytes to effectively perform this function. They are compartmentalized into distinct cellular domains that are populated exclusively by B cells or T cells with little extracellular matrix [92]. T cell responses are initiated in the T-cell zone of the lymph node within the paracortex and B cell responses are initiated in the B-cell zone known as the follicle [93]. The organization of the lymph node into distinct cellular regions is mainly controlled by chemokines [94]. Once antigen presenting cells display antigens to lymphocytes and provide the appropriate signals, T-cells differentiate into effector cells; either cytotoxic (CD8) T-cells or helper (CD4) T-cells and memory cells. B-cells, on the other hand, migrate to the border between the follicle and paracortex, where they present antigen-derived peptides to T helper cells. The antigen-specific B cells then receive signals from the helper T cells to proliferate, they undergo isotope switching and generate different classes of antibodies. Most effector cells then leave the lymph nodes and head towards the site of infection to eliminate the foreign antigens [95]. 

**Figure 3:**
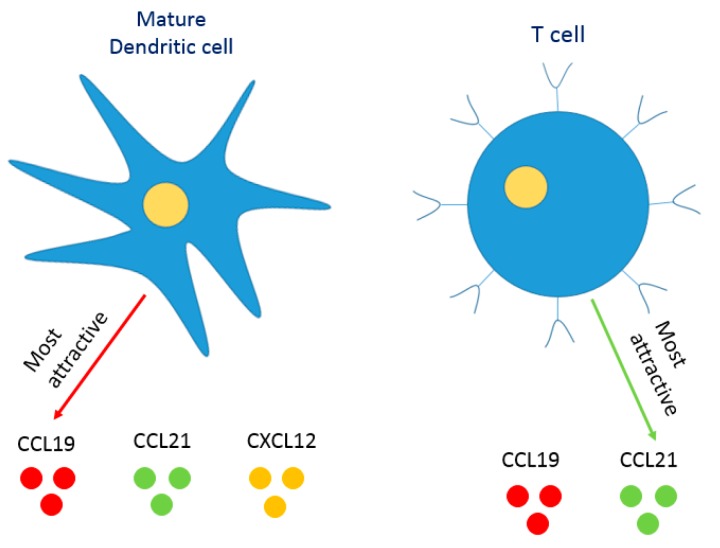
Response of different immune cells to chemokine gradients. Based on the work by [97,98], mature dendritic cells are much more attracted to the chemokine CCL19 than they are to the chemokines CCL21 or CXCL12. Interestingly, under physiological gradient.

The recreation of a lymph node in vitro is not an easy task, primarily because of its complex architecture and structural organization. Rosa and colleagues developed a microfluidic model of the paracortical region of the lymph node to examine the dynamics of interaction between dendritic cells and T-cells during flow at different shear stresses [93]. The model consists of a single channel with 2 inlets and 2 outlets whereby dendritic cells are cultured in the main channel and CD8+ cytotoxic T cells as well as CD4+ T helper cells are introduced into the device via the two inlets. The authors demonstrated that the duration and strength of interaction between T cells and dendritic cells vary depending on the amount of shear stress applied on the system and on the specific type of T-cell; antigen specific versus unspecific T cells and CD4+ helper T cells versus CD8+ cytotoxic T-cells. In order to achieve more physiological relevance and to model the migration of antigen presenting DCs from peripheral tissues to the lymph nodes and the subsequent interaction with T-cells, Mitra et al. developed a lymph node model to examine DC chemotaxis and the resulting T cell activation by migrated DCs [96]. The model consists of two layers of PDMS, a top layer being a chemotaxis compartment and a bottom layer being a T cell compartment. The DCs in the chemotaxis compartment are subjected to a chemokine gradient (CCL19) and their subsequent migration to the T cell compartment and their ability to activate T-cells are evaluated. T cell activation induced by DCs is assessed by measuring the level of calcium in T cells. The results indicate that mature DCs were respondent to the chemokine gradient, were capable of migrating to the T cell compartment and were able to activate the T-cells there, unlike immature DCs which did not respond to the chemokine gradient and did not effectively activate T-cells. In a similar chemokine study, Ricart et al. showed that mature DCs are respondent to the chemokines CCL19, CCL21 and CXCL12 but are much more attracted to the chemokine CCL19 than they are to the chemokines CCL21 or CXCL12 [97]. Interestingly, Nandagopal et al. showed that under physiological gradient conditions, human peripheral blood T cells chemotax to CCL21 but not to CCL19 [98]. Furthermore, T cells tend to migrate away from the CCL19 gradient in a uniform CCL21 background. Figure 3 summarizes the findings of Ricart et al. and Nadagapol et al. and shows the response of cells according to the present chemokines.

The above mentioned lymph node on chip devices successfully simulate several functions of the native lymph node, especially chemotaxis, the cellular response to chemokine gradients, where most of the lymph node studies were focused on this aspect. Surprisingly, only few of the existing studies assessed the lymph node’s ability to identify pathogens and fight infections. This aspect, in particular, is of vital importance in drug development and discovery to evaluate the toxicity of drug candidates to the immune components. Giese et al. developed an in vitro model of the lymph node and utilized it for immunological substance testing [99,100]. The model consists of 2 continuously perfused cell culture compartments separated by a double membrane. The authors introduced peripheral blood mononuclear cells (PBMCs) into the model along with commercial viral vaccine Hepatitis A (HavrixTM) and quantified immune cellular responses using cytokine secretion profiles. The results indicate that Havrix caused an initial increase in the secretion of TNF-alpha, a proinflammatory cytokine followed by a rapid decrease during the first days of culture. Subsequently, restimulation of immune cells with free antigens and with antigen-loaded DCs induced the release of TNF-alpha along with IL-6 and IL-10 which are T Helper Cell Type 2 promoting cytokines. However, the addition of an immune suppressive drug into the system significantly reduced cytokines secretion. These results reflect the ability of lymph node models to simulate the immunological outcomes in response to vaccines, pathogens and even pharmaceutical drugs. 

### 2.5. Spleen-on-Chip

Besides the lymph node, the spleen, a small soft organ located in the upper left side of the abdomen, behind the rib cage and stomach, is also a lymphoid organ that is a key component of the immune system. It acts as a filter purifying blood from viruses, bacteria, worn out or damaged red blood cells (RBCs) and other pathogens. It has two main regions called the red pulp and white pulp that are separated by an interface called the marginal zone [101]. The red pulp contains macrophages that engulf and destroy aged or damaged RBCs and subsequently regulate iron recycling. The marginal zone contains macrophages that destroy pathogens as well as dendritic cells that recognize and take up foreign antigens then migrate to the white pulp to present them to the lymphocytes resident there, which include B cells, CD4+ and CD8+ T cells [101,102]. The white pulp is structurally similar to a lymph node with specific B and T zones and is where antigen-specific adaptive immune responses are initiated. The spleen’s structural organization and cellular distribution are optimal for its filtration function; the blood micro-circulates through filtration beds in the spleen’s red pulp where its hematocrit is increased [103]. This facilitates the removal of damaged RBCs by macrophages and the recognition of antigens by dendritic cells then their subsequent presentation to lymphocytes. To further ensure the elimination of pathogens and damaged RBCs, the blood is allowed to travel only in one direction through certain interendothelial slits to reach the circulatory system [103]. 

Despite its importance in immunity, only few attempts have been made to model the spleen in vitro, perhaps because survival without a spleen is possible. Rigat-Brugarolas and colleagues developed a microfluidic device that mimics the physical properties and hydrodynamic forces of the splenon, the spleen’s basic functional unit [103]. The device consists of two channels, one that supports fast fluid flow and another that supports slow fluid flow which is analogous to the microcirculation in the red pulp. In the slow channel, the blood flows through a pillar matrix where the hematocrit of blood is increased and then through constricted channels that force the cells to deform. The extent of deformation varies according to cell healthiness and provides a mean to differentiate between healthy and unhealthy cells [104]. The device’s filtration ability has been evaluated with healthy and malaria-infected RBCs. Despite demonstrating the potential of a spleen-on-chip for differentiating between healthy and infected cells, there is room for improvement specifically with regards to the implementation of the removal or destruction process of damaged RBCs and of other pathogens by macrophages and DCs. Ingber and co-workers developed a microfluidic device that mimics both the blood filtering and cleansing function of spleen thus, acting as an external filtration system analogous to hemodialysis machine [105]. Blood is mixed with magnetic nanobeads coated with an engineered antibody (mannose-binding lectin) that binds to polysaccharides that are found on the surface of more than ninety types of antigens and toxins. Magnets pull the bead-bound pathogens and toxins from the blood. The clean blood is then returned back to the individual. The spleen-on-chip device was shown to efficiently remove multiple Gram-negative and Gram-positive bacteria, fungi and endotoxins from whole human blood. Although this spleen-on-chip device is intended for sepsis therapy, its use can be extended to drug development and discovery as well as to personalized medicine by accelerating the process of pathogen identification and drug susceptibility determination, especially since the modified protein can bind to both live and dead pathogens. 

### 2.6. Bone Marrow-on-Chip

All immune cells, including the lymphocytes of the adaptive immune system, originate in the bone marrow and some also mature there. Once ready, these cells move into the bloodstream and circulate through the lymph nodes and other lymphoid organs to perform their defensive functions. This makes the bone marrow an important component of the immune system, if not the most important. Any infection or disease that affects the bone marrow compromises the ability of the immune system to eradicate the body from pathogens, leading to serious diseases or death. 

The bone marrow is a complex organ containing both hematopoietic and non-hematopoietic cells surrounded by a shell of vascularized and innervated bone [106]. Hematopoietic stem cells, which give rise to immune cells, reside in distinct microenvironments within the bone marrow, known as niches. These niches provide the hematopoietic stem cells with various regulatory signals necessary for their maintenance, and host the generation of healthy mature blood cells in appropriate numbers throughout an individual’s life time [107]. 

The presence of various niches within the bone marrow, each having different components and different functional roles in hematopoiesis, make it difficult to reproduce the complex microenvironment of the bone marrow in vitro [88,108]. Nevertheless, Torisawa et al. combined both in vivo tissue engineering and in vitro microfluidic approaches to develop a bone marrow on chip device that replicates the hematopoietic niche physiology in vitro [109]. To do this, they fabricated a microfluidic PDMS device with a cylindrical cavity sealed at one end, and filled the cavity with Type I collagen gel containing bone-inducing demineralized bone powder as well as bone morphogenetic proteins then implanted the device subcutaneously in the back of a mouse. The aim is for new bone, containing a marrow, to form in the cavity and be later isolated for further culture in a similar perfused PDMS device but in vitro. The authors showed that new bone encasing a marrow compartment formed inside the device within eight weeks after subcutaneous implantation. The obtained bone was also able to maintain functional hematopoietic microenvironments even when transferred to the in vitro perfused microfluidic device. Torisawa et al. later demonstrated the ability of the bone marrow on chip to continuously produce blood cells in vitro, both leukocytes and RBCs, and modeled its response to radiation countermeasure drugs [110]. When the bone marrow chip was exposed to gamma radiation, the production of leukocytes decreased significantly. However, when the chip was subsequently treated with two potential therapeutics, the number of hematopoietic stem cells and myeloid cells increased significantly. More recently, Sieber et al. developed a pure in vitro bone marrow-on chip that sustains the culture and multiplication of multipotent hematopoietic stem and progenitor cells [111]. It is based on a hydroxyapatite coated zirconium oxide ceramic scaffold and was shown to sustain successful hematopoietic stem and progenitor cells culture for up to 28 days in a microfluidic environment with the bone marrow niche remaining intact. Furthermore, in order to demonstrate how such organ on chip models could be utilized for studying pathogenesis and for drug discovery, Bruce et al. developed a bone marrow on chip to study Acute Lymphoblastic Leukemia and to test the effectiveness of an antimetabolite chemotherapeutic drug, cytarabine [40]. Human bone marrow stromal cells, osteoblasts and human leukemic cells representative of an aggressive phenotype were encapsulated in 3-D collagen matrix and cultured within a microfluidic platform consisting of 4 microfluidic channels with a single inlet and a single outlet. The cells in the microfluidic platform were subjected to cytarabine containing culture medium and the effect of cytarabine on 2D and 3D systems in both static and dynamic conditions was compared. The results show that in both the 3-D dynamic as well as 3-D static models, coordinated cell-cell interactions between the 3 cell types were present while in 2-D static model, these interactions were absent. In addition, the study showed decreased leukemic cells sensitivity towards chemotherapeutic drug in 3-D tri-culture models compared to the 2-D models. These results shed the light on the importance of understanding the role of the microenvironment in controlling cellular behavior and the necessity to develop platforms more accurately simulating in vivo conditions for evaluating drug efficiency and toxicity at an early stage of drug development.

### 2.7. Lymphatic Vessel-on-Chip

Lymphatic vessels collect the substances that leak from the vascular system such as plasma, proteins, antigen presenting cells and pathogens and return them back to the circulatory system. Collectively, the substances picked up by the lymphatic vessels make up the lymph fluid. As lymph fluid flows through the lymphatic vessels, it passes through the lymph nodes, where its constituents are screened by lymphocytes. Once a foreign antigen is detected, lymphocytes proliferate and differentiate into effectors cells initiating an immune response. Thus, lymphatic vessels are crucial for immunity, as they mediate the communication between the tissues of the body and lymphoid organs. Recently, the view towards lymphatic vessels has shifted from simply regarding lymphatic vessels as passive conduits of lymph fluid and immune cells, to regarding them as active, tissue-specific participants in major physiological and pathophysiological processes [112]. 

A lymphatic vessel on-chip platform should facilitate studies aimed at understanding the mechanism by which substances exit the circulatory system and enter the lymphatic system. More relevant to the immune context, lymphatic vessel on-chip should help in understanding how antigen-presenting cells and lymphocytes leave an inflamed tissue and enter into nearby afferent lymphatics. This process, in fact, is vital for the initiation and maintenance of an immune response [113]. Sato et al. developed a microfluidic model of microcirculation containing both blood and lymphatic vessels [42]. The model consists of upper and lower channels that are separated by a porous membrane onto which blood vascular endothelial cells (BECs) and lymphatic endothelial cells (LECs) are co-cultured back-to-back. The authors showed that the flow established within the device promoted the formation of endothelial cell-cell junctions with detectable claudin-5 and VE-cadherin production. In addition, they demonstrated that the addition of histamine resulted in an increase in the vascular permeability and induced changes in the localization of tight and adherens junction-associated proteins. It would be interesting to assess how the addition of a pharmaceutical drugs affect the vascular permeability as well as the cellular junctions and associated proteins. 

### 2.8. Other Relevant Immune-on-Chip Platforms

Besides the discussed immune organs-on-chip, other on-chip platforms have been developed to facilitate investigations into immune physiology and disease mechanisms as well as to aid in the process of drug development. These include inflammation-on-chip, cancer-on-chip and immunotherapy-on-chip [114,115,116]. Inflammation-on-chip models enabled the precise monitoring of immune cells in the presence of relevant chemical gradients and microbe-like particles [114,117]. In addition, they assisted in examining immune cell interactions such as neutrophil–monocyte interactions and in quantifying cellular biochemical secretion profiles [114,118]. Furthermore, certain blood brain barrier-on-chip models have been developed and utilized to assess the role of neuro-inflammation in the initiation and progression of degenerative diseases such as Alzheimer’s disease and to study antibody transport to the central nervous system [119,120]. On the other hand, cancer-on–chip models aided in understanding the complex interactions between immune and cancer cells [121,122]. These models also unraveled subtle differences in the motility and antitumor activity of lymphocytes depending on local conditions within tumor microenvironment such as local oxygen levels and inflammatory cytokine gradients [123]. Additionally, immunotherapy-on-chip models recreated immune-cancer microenvironments and assessed the efficacy of immunotherapeutic approaches [116].

The field of on-chip platforms for biological applications remains quite broad and contains many other organ-on-chip models that do not specifically examine immune functions such as adipose-on-chip, eye-on-chip and placenta-on-chip models [124,125,126,127]. It is an interesting scientific study to assess the immune competence of such tissues and organs especially since it was recently shown that they play significant roles in maintaining immune homeostasis [128,129,130,131]. In addition, the eye and placenta in particular, exhibit the so called immune privilege which enables them to tolerate the introduction of antigens without eliciting an inflammatory immune response [132,133]. 

## 3. Towards an Integrated Immune System on Chip

Most existing organ-on chip platforms including those of the immune system replicate a single organ only. Table 1 summarizes available organs-on-chip that simulate immune organs and discusses their relevance to drug development, i.e., how these immune organs contribute to tolerance towards newly developed drugs. Such organ-on-chip models that replicate a single organ do not permit investigations on a drug’s systemic effect [7]. However, there has been a growing interest in developing co-culture and multiple organ models known as microphysiological systems or body-on-a-chip devices to better predict systemic effects of drugs [134,135]. Such multi-organ devices comprise various cell types cultured in separate chambers within a single chip whereby each chamber is representative of a different organ. The chambers are connected via channels in a particular sequence based on the nature of interactions between an organ and another in vivo [7]. This allows investigations on the effects of a drug on multiple organs simultaneously.

Although several multiorgan devices have been developed for systemic drug testing in vitro [136,137,138,139,140], there is no existent model that incorporates all immune organs on a single chip. In an ideal in vitro immune system on chip, a drug candidate would be initially introduced either into the skin equivalent resembling subcutaneous injection or into the gut equivalent resembling oral administration. Subsequently, the drug will be transported through a vascular equivalent and a lymphatic equivalent to the spleen and lymph node respectively. The interaction of the drug candidate with the immune components at each organ would be accurately evaluated and quantified. Such systemic assessment of drug toxicity to the immune system better predicts the performance of drugs during clinical trials especially if cells incorporated into the in vitro immune system on chip are primary cells derived from humans. If available, such immune model would facilitate the mechanistic understanding of our immune physiology to drugs and thus contribute to reducing both the high cost and the high attrition rate associated with drug development especially since toxicity to the immune system is a major contributor to drug failure in clinical trials [2]. In addition, it will help progress our understanding of disease physiology and mechanisms as well as of infections and autoimmunity.

## 4. Challenges

Despite the great potential of the organ-on-chip technology, key biological and technical challenges remain. These include achieving a proper scale in terms of organ size and cell number, attaining architectural complexity of the human tissues and organs in vitro and in a miniaturized manner, developing a universal perfusion medium suitable for multiple cell types within the same organ or within different organs connected together and utilizing primary human cells rather than cancerous cell lines [7,141]. Furthermore, especially for drug development applications, the material utilized to fabricate the microfluidic device needs not to influence cellular behavior or response. Currently, polydimethylsiloxane (PDMS) is the standard material used for fabrication. However, PDMS in itself is highly lipophilic thus, can bind to molecules in the perfusion medium or to introduced drugs. Therefore, researchers are exploring different modifications to PDMS that can reduce or eliminate these interactions such as coating it with inert polymers or treating it with gas plasma or UV light [142]. Furthermore, when connecting organ-on-chips to microfluidic tubing for system perfusion, bubbles can be easily introduced which can disrupt cell culture and compromise system sterility [141]. 

Besides the challenges associated with developing and using organ-on chips, the adoption of such technology for drug development and testing presents another barrier. The technology needs to be extensively validated before it can be approved by the FDA as an efficient preclinical method of testing newly developed drugs. This would require strong collaboration between researchers, regulators and the industry.

## Figures and Tables

**Table 1:** Summary of available immune-organs-on-chip.

Organ	Simulated Feature/s	Relevance to Drug development	Reference
Skin	Barrier Function	The skin is a site of administration of some drugs Skin is an active immune organ that reacts to pathogens and foreign substances including drugs Immune rejection to newly developed drugs can be initiated in skin, thus, models assessing drug-skin immune interaction are desirable	[48,49]
	Vascularization		[39,51,52]
	Air-to-liquid interface		[49,50]
	Absorption		[39]
	Inflammation and Edema		[39]
	Immune Competence		[53]
Gut	Epithelial Barrier function	The Gut is a site of administration of some drugs (oral administration) The Gut contains various immune components that react to pathogens and foreign substances including drugs Immune rejection to newly developed drugs can be initiated in gut, thus, models assessing drug-gut immune interaction are desirable	[60]
	Peristalsis motion		[61,65,66]
	Microbial interface		[61,66,68]
	Substance Transport		[61]
	Gut viral infection		[41]
	Villi		[61,65,66,67]
	Immune Competence		[66]
Liver	Liver sinusoid	The liver has been shown to play key roles in immunity containing the body’s largest population of macrophages (Kupffer cells) Drug toxicity to the liver is the most common cause for the discontinuation of clinical trials on a drug, as well as the most common reason for an approved withdrawal of a drug from the marketplace [89] Thus, interactions between liver immune components and drugs should be assessed	[74,80,86,87]
	Substance Transport		[75,79,80,83,84]
	Metabolism		[77,80,84]
	Bile Canaliculi		[83]
	Secretion of liver specific products (urea and albumin)		[82,84,86]
	Hepatocyte-endothelial interface		[74,78,81,87]
	Hepatocyte-fibroblast interaction		[82]
	Hepatocyte-stellate cell interaction		[85]
	Hepatitis B virus Replication		[87]
	Drug toxicity testing		[77,80,84]
Lymph node	Chemotaxis	Lymph node is the site of immune coordination in the body Adaptive immune responses against newly developed drugs are initiated in the lymph node thus, lymph node models can be utilized to modulate response of immune cells to drugs	[97,98]
	Immune cell interactions		[93,96]
	Response to vaccines and drugs		[99,100]
Spleen	Blood filtration	Spleen filters the blood and removes pathogens and other foreign substances including pharmaceutical drugs Models assessing the cleansing process of the spleen which involves immune components such as macrophages are highly desirable especially for assessing how drugs are eliminated	[103,105]
	Blood cleansing		[105]
Bone Marrow	Hematopoietic Niche Formation	The bone marrow is the site of production of all immune cells as well as the site of selection and maturation of B lymphocytes Models examining the development of lymphocytes within bone marrow and investigating the priming process of B-cells as well as the specific cellular mechanism of recognizing and responding to an antigen can aid in developing drugs that are less toxic to the immune system	[40,110]
	Blood and immune cell production		[109,110]
	Responsiveness to drugs		[109,111]
Lymphatic vessels	Microcirculation	Exchange of substances between circulatory and lymphatic system occur through lymphatic vessels therefore, models examining how pharmaceutical drugs leave bloodstream and enter into nearby afferent lymphatics are highly desirable	[42]
